# A CDST Perspective on Variability in Foreign Language Learners’ Listening Development

**DOI:** 10.3389/fpsyg.2021.601962

**Published:** 2021-02-03

**Authors:** Pengyun Chang, Lawrence Jun Zhang

**Affiliations:** ^1^School of Foreign Languages and Cultures, Chongqing University, Chongqing, China; ^2^Faculty of Education and Social Work, University of Auckland, Auckland, New Zealand

**Keywords:** Complex Dynamic Systems Theory (CDST), inter-individual and intra-individual variability, EFL listening, English as a foreign (second) language, foreign language learning and teaching

## Abstract

Within a Complex Dynamic Systems Theory (CDST) framework, this longitudinal qualitative study explored the complex patterns and identified the degree of variability in three learners’ developmental process. Learners’ listening performance was tracked and examined every 6 weeks, followed by retrospective interviews and self-reflections every 7 weeks over the 43-month span. A series of CDST techniques were adopted for data analysis, including using min–max graphs to trace the minimum and maximum scores on the EFL learners’ listening developmental indices over time. Monte-Carlo and Loess smoothing analyses were applied to gauge for degrees of variability. The results suggest that: (1) Min–max graphs and smoothed Loess curves depict flux developmental processes of learners’ L2 listening; (2) learners differed from each other in the degree of inter-individual variability in their listening developmental trajectory; and (3) occurrence of unanticipated patterns confirm that learners demonstrated personalized intra-individual variability within their unique listening developmental process. Results indicate that variability is a pattern characteristic of CDST both between and within individuals, and inform us about how Chinese EFL listeners’ language develops. We conclude by discussing the implications for researchers and practitioners who are concerned with learners’ developmental trajectories and unexpected changing patterns in the process of foreign language learning.

## Introduction

The trend toward internationalized education has been accelerating rapidly worldwide over the past decades. This is particularly the case with regard to Chinese learners of English as a second/foreign language (L2), who form a significant proportion of the overseas learning population and face both opportunities and challenges. For example, understanding and communicating with others in English is a necessary and vital skill for Chinese learners today, and thus there has been a steady increase in L2 learning research not only in China but also in other contexts where L2 learners form a substantial proportion, concentrating on improving learners’ communicating ability (e.g., L2 listening capacity) using different theoretical frameworks (e.g., the cognitive-interactionist perspective and sociocultural theory), in different tutorial settings (e.g., classroom-based and conference-based), providing various types of instructions (e.g., task-based and strategy training) for learners at various language proficiency levels. The existing body of L2 research has heightened the importance of input and its effect on learners’ listening results. However, the complexity of the learning process and L2 learners’ variability in the development of L2 listening remain under-explored.

Complex Dynamic Systems Theory (CDST) as a theory seeks to explain language use and development phenomena in terms of emergent systems that are complex, interconnected, dynamic, self-organizing, context-dependent, open, adaptive and non-linear (Larsen-Freeman, 2019). Variability in second language development (SLD), from the CDST perspective, concerns the developmental dynamics that have traditionally been overlooked. How learners differ from each other in their learning process? Is it possible to visualize learners’ individual learning trajectories? What is the uniqueness of each learner in their language developmental process? Adopting a qualitative multiple-case study approach, this study was conducted to fill the research gap by tracking three advanced university-level English learners (Chinese as their L1) for 43 months, aiming to explore the variability demonstrated in the individual trajectories and dynamic processes of their L2 listening development from a CDST perspective.

## Literature Review

### Complex Dynamic Systems Theory (CDST)

Complex Dynamic Systems Theory has been proposed and used to describe developmental processes (e.g., van Geert, 2008; Spoelman and Verspoor, 2010; Verspoor et al., 2017; Larsen-Freeman, 2018, 2019; Lowie et al., 2018) and variability (e.g., van Geert and van Dijk, 2002; Larsen-Freeman, 2006, 2017; Polat and Kim, 2014; Lowie and Verspoor, 2015, 2018) in L2 learning in a number of recent SLA studies, proposing non-linear development of overweight cause-effect relationships, and emphasizing that disordered details and dynamic changes can be indicators of development (Larsen-Freeman and Cameron, 2008; Verspoor et al., 2008, 2017; van Dijk et al., 2011).

From a CDST perspective, language, language acquisition and attrition are characteristics of development as progress, which are much more intricate, complex, and even unpredictable than what a linear position would allow. Thus, the term, SLD is preferred rather than second language acquisition (SLA) in recent studies, on the basis of considering language learning as a non-linear and complex dynamic process (De Bot and Larsen-Freeman, 2011; Larsen-Freeman, 2015). A group of L2 studies were conducted to explore learners’ writing development (Larsen-Freeman, 2006; Verspoor et al., 2008; Spoelman and Verspoor, 2010; Hou, 2017). Other linguistic features including vocabulary development (Zheng, 2011; Zheng and Feng, 2017), learners’ strategy use and listening performance (Dong, 2016), learner agency (Mercer, 2011), chunks learning (Verspoor and Smiskova, 2012) and English speech (Polat and Kim, 2014) have also been examined by an increasing number of researchers. The findings of these studies confirm that language learning has interconnecting and self-organizing systems that co-adapt, and which may display sudden discontinuities and the emergence of new modes and behaviors (Larsen-Freeman and Cameron, 2008).

### Variability in Language Development

Accordingly, longitudinal studies were conducted from a CDST perspective, finding that variability and variations existed among similar learners who were exposed to similar circumstances (e.g., Larsen-Freeman, 2006; Chan et al., 2015; Chang and Zhang, 2020). In the CDST framework, variability is a central element in the process of language development; it is not only an extrinsic property differentiating learners, but “a harbinger of change” (Thelen and Smith, 1994, p. 342). In particular, differences that are identified between learners are viewed as inter-individual variability, and seen as a key point to differentiate levels and make comparisons (Verspoor et al., 2008). Variability has been viewed also as an intrinsic property of a self-organizing, developing system, where learners have their own paths (cf. De Bot et al., 2005). It is assumed that variability can help us discover developmental patterns that otherwise would remain hidden. Essential to this dynamic approach, differences demonstrated in each learner’s developmental process are viewed as intra-individual variability, providing a stimulus for growth and recognizing learners’ uniqueness in L2 learning. This might be a phase shift or a developmental jump, which are important features that should be treated as data and analyzed within the dynamic system (Verspoor et al., 2008; Larsen-Freeman, 2020).

### Studies on L2 Listening From the CDST Perspective

Listening, characterized by its transient nature brings challenges and difficulty to scholars in terms of exploration and analysis. The same is true of L2 listening. To date, existing L2 listening studies from the CDST perspective have mainly focused on theoretical discussion and the identification of potential affecting factors – for example, in the dynamic metacognitive model proposed by Zhang and Zhang (2013), listening strategies were widely accepted as effective contributors to enhancing students’ listening performance (Cross, 2010; Zhang, 2010; Vandergrift and Baker, 2015). Within the CDST framework, Qiu and Huang (2012) proposed a dynamic image schema model and explored its effects on EFL learners’ systematic improvement in listening comprehension.

Despite extensive findings, most of the previous studies have treated listening passively, and were mainly focused on comparisons between pre-test and post-test listening scores in investigating the listeners’ proficiency change and the exploration of possible influential elements in predicting listeners’ performance level. Little is known about how learners develop through individually owned trajectories. Thus, this study sets out to explore L2 listening from a longitudinal CDST perspective. It is hoped that the results will provide insight not only into an individual’s stages of SLD, but more specifically into the developmental process of L2 learning (Verspoor et al., 2008, 2017; Larsen-Freeman, 2018). This study aims to view listening performance as an effective indicator of L2 listening capacity, to explore the developmental processes of learners and their differences, as well as examine the inter-individual as well as the intra-individual variability within their own developmental trajectories.

### Research Questions

Seeking to elucidate the above aspects by tracking three advanced learners’ listening development, tracing their listening performance as well as observing and analyzing their degrees of variability, it intends to address the following questions:

(1) What are the developmental processes of learners’ listening performance?
(2) What is the inter-individual variability of learners’ listening performance?
(3) What is the intra-individual variability, and what patterns in variability did the EFL learners demonstrate over time?

## Materials and Methods

In order to observe changes in EFL learners’ listening development and examine their intra-individual variability, a longitudinal design with repeated measurements was adopted in this study.

### Participants

Five learners (four females and one male, 20 years old on average) were invited to participate in this longitudinal study from 2016 to 2019. They were second-year students, majoring in statistics. All of them were recruited on a voluntary basis from a university in Northern China, and all of them experienced an average of 6 h of English classes per week. Meanwhile, they also spent time on various English practices or after-class activities, ranging from 1 h to 10 h per week. Two of the five participants dropped out for personal reasons at the end of the first and second years of the experiment. Anonymous names were used in this paper.

### Instruments

This longitudinal study adopted various measures for data collection, including listening tests, retrospective interviews, and self-reflections to triangulate learners’ learning processes and their inter-and intra-individual variability.

#### Listening Tests

The listening tests used to assess participants’ listening performance were selected from The International English Language Testing System (IELTS), which is widely recognized as a reliable means of assessing the language ability of candidates who need to study or work where English is the language of communication. IELTS is owned by three partners, the University of Cambridge ESOL Examinations, the British Council and IDP Education Pty Limited^1^. Thus, the reliability and validity of the IELTS tests are well established (IELTS, 2017). The IELTS listening test contains four independent sections, each with 10 questions (see Supplementary Appendix I as an example). The first two sections are concerned with social needs (e.g., make reservations, book tickets or inquiries of library policies) and the final two sections are concerned with situations related to educational or training contexts (e.g., biology lectures, academic conferences or preparations for field trips), which are presented by conversations and monologs. Thus, topic variation is the first reason IELTS listening tests were used in the current study. Secondly, a variety of question types is used in the IELTS listening tests, including: multiple choice, matching, plan/map/diagram labeling, completing tables or diagrams, summarizing, sentence completion and short-answer questions. In order to ensure test consistency, four question types were selected and adopted in the current listening test, namely multiple choice, matching, single word cloze, and phrase or sentential cloze questions. Participants listened to the recording once and answered the questions immediately. Given that the test papers were all designed following the standard IELTS listening tests, it is assumed that the test versions were homogeneous in terms of their level of difficulty. During the 43-month longitudinal study the listening test was arranged every 6 weeks, 30 times in total. The maximum possible score for the listening test was 40 points, with each section consisting of 10 questions contributing 1 point each.

#### Retrospective Interviews

Rather than predicting the future from the present, retrospective interviews (see Supplementary Appendix II) use present evidence to look back in someone’s developmental history, to see whether the present state of affairs may be explained by the past (Dörnyei et al., 2015). In other words, retrodiction requires backward inference from present data. This study employed retrospective interviews with the expectation of finding evidence of past events or patterns which may explain learners’ present listening performance (Larsen-Freeman and Cameron, 2008; Chan et al., 2015). According to De Bot (2015), timescales can be arranged from 1 month to 1 year if the time window is around 2 years. Thus, altogether 24 timescales were arranged every 7 weeks, as the current study lasted for nearly 3.5 years. The participants were invited to recall their learning experiences over the preceding 7 weeks in terms of English classes, listening activities, tests and their reflections. As approved by the participants, the interviews were recorded and then transcribed for analysis.

#### Self-Reflections

Participants were also invited to provide written reflections every 7 weeks (24 times altogether) during the 43-month span. Prompts were provided by the researcher for learners to record their learning procedures, listening difficulties and feedback, as well as to share their learning experiences, materials, feelings and anything that might affect their listening development during the learning process (see Supplementary Appendix III).

### Data Collection Procedures

First of all, consent forms were received from all participants before the start of data collection. Data collection for this study consisted of listening measurements, the retrospective interviews and self-reflections, which were scheduled about 43 months from 2015 to 2019. The listening assessment was administered every 6 weeks to trace the participants’ listening performance, lasting for 40 min each time. All research sessions were conducted either on the main campus of the participants’ university or completed online through internet. During the following week, namely, every 7 weeks, the retrospective interviews were arranged and self-reflections were collected to investigate the details of participants’ listening development, which were all completed online through QQ and WeChat, two online social media platforms widely used in China and elsewhere among the Chinese diaspora.

### Data Analysis

#### Min–Max Graphs

The moving min–max graph depicts the moving minima, maxima, and observed values of the variables, and highlights “the general pattern of variability, while keeping the raw data visible” (Verspoor et al., 2011, p. 75). Thus, it was employed to observing the overall developmental process of students’ listening performance, within which the temporary changes and the degree of variation in students’ listening performance can also be detected through the moving min–max graph. As a descriptive method, the moving min–max graph is also used to spotlight variability changes as well as patterns in learners’ listening developmental trajectory. Aiming of presenting a detailed picture of the developmental patterns, three consecutive measurement points was chosen as the predetermined moving window span in this study (van Geert and van Dijk, 2002; Verspoor et al., 2011).

#### Monte Carlo Analysis

The Monte Carlo (random permutation) technique (van Geert et al., 2012), particularly comparative analysis, was used to calculate whether there was any statistical significance in the differences observed in the three learners’ developmental trajectories. Because of the sensitivity of the listening system, there may have been unanticipated patterns that might trigger a turning point or cause the system to veer in a different direction. Looking for these unanticipated patterns is important because they can initiate a phase shift in a learner’s language resources, often resulting in a bifurcation (Evans, 2018), a characteristic pattern in a complex dynamic system (Larsen-Freeman, 2020). Monte Carlo analysis, specifically with the between-session variability measurement, focuses on exploring the statistical coincidence of the presence of each learner’s unexpected changes in their developmental trajectory. Through resampling the original data and shuffle it for 5000 times, the expected *p*-value will be reliable for this statistical technique is appropriate for the observations in this study (van Geert et al., 2012). In addition, evidence may be found in frequently occurring patterns in longitudinal corpora of the learners’ self-report journals and semi-structured retrospective interviews, which can provide useful signposts for tracing the trajectory of a dynamic system.

#### Smoothed Loess Curves

The current study aims to explore the general listening development and reveal the underlying developmental trends in learners’ listening during the 43 months of the project. Thus, with the capacity to model complex and uncertain processes in developmental patterns, the PTS LOESS Smoothing Utility (Peltier, 2009) was employed to provide smoothed Loess curves. This is an efficient exploratory analytical tool, achieved by “weighting the data proportional to their distance from the middle of the window” (Bassano and Van Geert, 2007, p. 595). The purpose of smoothing is to “sketch” the general trend of the data and leave out many of the irregularities of the actual data. Smoothers are therefore very well suited for representing a possible direction, namely, the listening developmental trajectory in this current work. Following Peltier (2009), the smoothing parameter alpha was set at 0.33, accordingly the moving window was nine observation points, to allow the smoothed Loess curves to better display general patterns while demonstrating the local patterns of variation.

#### Interpretive Phenomenological Analysis

In working with the qualitative data in this study, interpretive phenomenological analysis (IPA) was employed. IPA is one of several allied phenomenological analytical techniques, and its central concern is the subjective conscious experiences of individuals (Eatough et al., 2008). The first stage involved reading through the transcripts several times and making notes about interesting features in the left-hand margin of the document. In the second stage the transcripts were re-read, this time with the aim of incorporating theoretical abstractions so as to transform the initial notes and ideas into relevant themes, such as intra-individual variability patterns in the current work. These concepts and ideas were noted in the right-hand margin. In the third stage, connections were made between the different ideas developed in stage two, and a number of central themes emerged. The final IPA stage that normally involves the creation of a summary of themes was not carried out.

## Results and Discussion

This section presents the results of our analysis of each of the three learners’ listening performance, aiming to explore and confirm the inter-individual variability in their listening development over the 43-month period.

### Inter-Individual Variability

#### Non-linearity of Learners’ Listening Development

**FIGURE 1:**
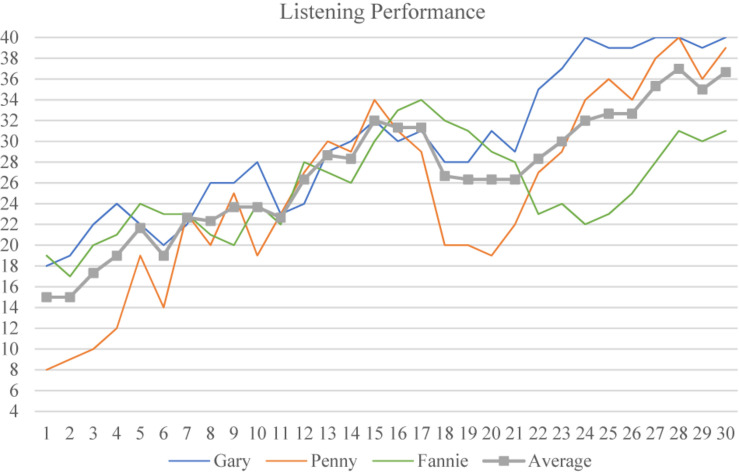
The developmental trajectory of learners’ listening performance.

The developmental trajectory of the three learners’ individual listening performance and their average (the gray line) are illustrated in Figure 1. As can be observed from their average, the students’ listening performance was accompanied by salient fluctuations in the 30 times measurements (30-Ms) over the 43-month span. In other words, the trajectory of the learner’s listening performance was characterized by an alternation of progress and regression instead of a linear developmental path.

Generally, it seems there are three stages in learners’ listening development. The first stage was from M-1 to M-10. During this period, Gary seemed to perform better than the other two participants as his performance was mostly above the average, although downward trends were also detected. Penny also seemed to experience significant increases but also decreases in her listening performance, and managed to exceed the average level at M-9. Similarly, Fannie experienced fluctuations with a smoother process, demonstrating less variation in her performance during this first stage.

In the second stage, M-11 to M-15, the three learners reached peaks in their listening measurement (Fannie achieved hers at M-17) as their average scores increased gradually. Moreover, the overlapping lines in Figure 1 indicate that there may be less variability between the three learners’ listening performances during this stage.

The third stage from M-16 to M-20 witnessed sudden regressions in the learners’ listening performance, during which Penny demonstrated the greatest fluctuations and her listening performance fell below the average level again. Gary and Fannie also experienced different levels of decrease. It should be noted that even if descending trends were observed, the three participants were still performing better than in the first stage.

Gary and Penny’s listening performance bounced back in the final stage, M-21 to M-30, during which their performance climbed steadily and achieved an almost perfect score around M-27. It seems they reached a new summit and entered a comparatively stable stage in their listening development. These results confirm Siegler (2006) report that learners do not progress neatly when acquiring a skill, but with periods of progression and regression which tend to be greatest during periods of rapid learning.

Fannie, however, experienced a longer period of regression than her counterparts in the final stage. During this stage Fannie’s listening performance was below average, and neither caught up with Gary and Penny, nor surpassed her best performance (at M-17), although her listening trajectory demonstrated an ascending trend from M-24 onward. Our findings suggest that learners experience variations as well as stable patterns in their listening development over a longitudinal observation, and demonstrate non-linear developmental trajectories with individual features that are worth of further exploration (Larsen-Freeman, 2006).

#### Inter-Individual Differences in Learners’ Listening Performance

**FIGURE 2:**
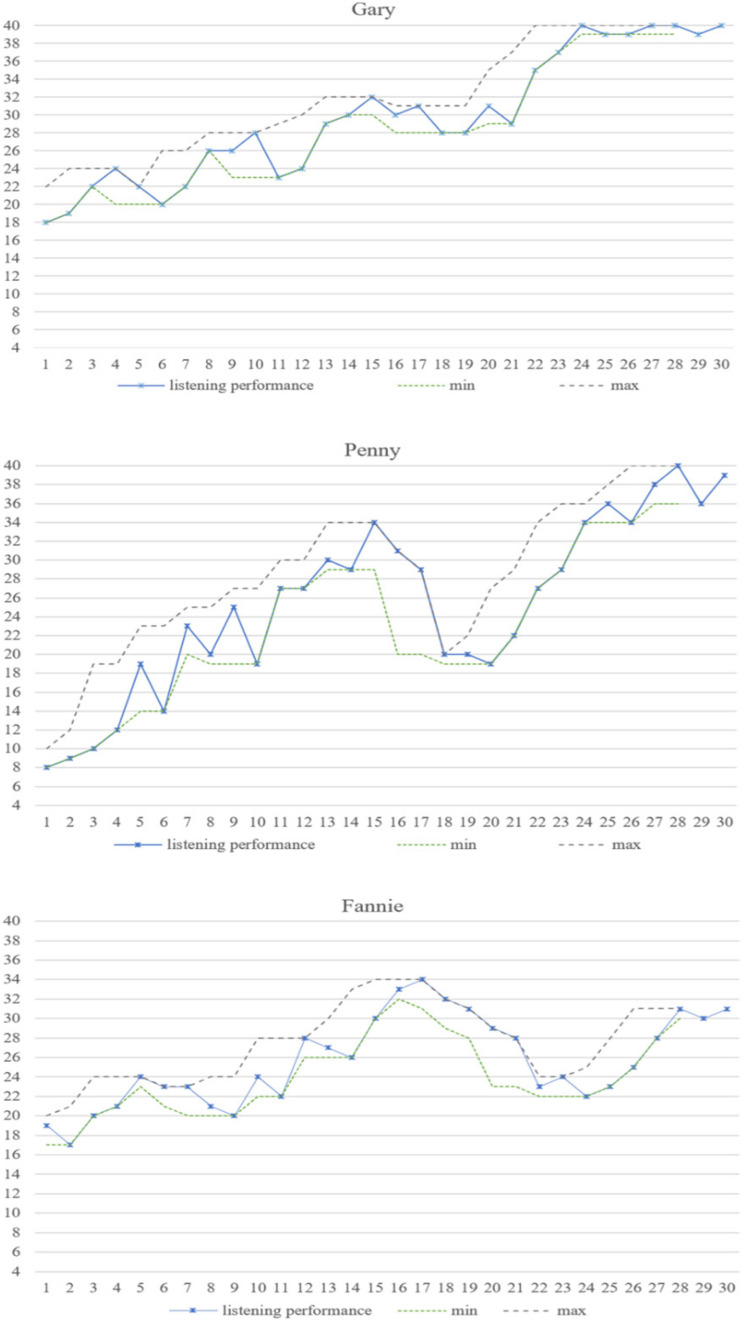
The developmental trajectory and moving min–max graphs of learners’ listening performance.

This section will go further to analyze the general trends in the learners’ listening development, as well as the fluctuations they demonstrated, through min–max graphs across the raw data of their listening performance. The paths of the learners’ listening trajectory and the min–max values are illustrated in Figure 2.

As shown in Figure 2, the learners’ listening trajectories followed noticeably non-linear patterns (see also Figure 1). The min–max graphs in Figure 2 further depict the varying degrees of their listening variability, which were characterized by the bandwidth of the min–max graphs – namely, the larger the bandwidth of scores, the higher the degree of variability (Larsen-Freeman, 2006). Thus, the three learners experienced more fluctuations up to M-15, but fewer from M-16 onward. Because of the bandwidth of Penny’s results, we assumed Penny was more variable than Gary and Fannie in her listening development. In order to test this and explore whether there were statistical differences among three learners’ listening performances, specifically their inter-individual variability, we used Monte Carlo comparative analysis to compare their listening results with respect to each other (van Dijk et al., 2011).

We set up a resampling model based on the original raw data, and randomly reshuffled it 5000 times (as a remedy for low numbers of data points), and a conventional significance level (*p* ≤ 0.05) was used for this analysis (see Hood, 2009, for details). The results showed that significant differences were identified between Penny and Gary’s listening performance (*p* = 0.002, *p* < 0.05), as well as between Penny and Fannie’s listening results (*p* = 0.05). This confirmed our assumption that Penny showed more divergent developmental patterns, or her trajectory was more variable, than either Gary or Fannie. Furthermore, a statistical significance level of *p* = 0.09 was found between Gary’s and Fannie’s listening performances, which did not reach the significance threshold and indicates that there are no significant differences in their developmental trajectory.

Thus, inter-individually, participants’ listening developmental trajectories vary in different degrees, depicted by the bandwidth displayed in the min–max graphs. Specific statistical tests further confirmed that inter-individual differences also existed in their learning trajectories in similar circumstances, as discussed below.

#### Significant Variations in Inter-Individual Variability

**FIGURE 3:**
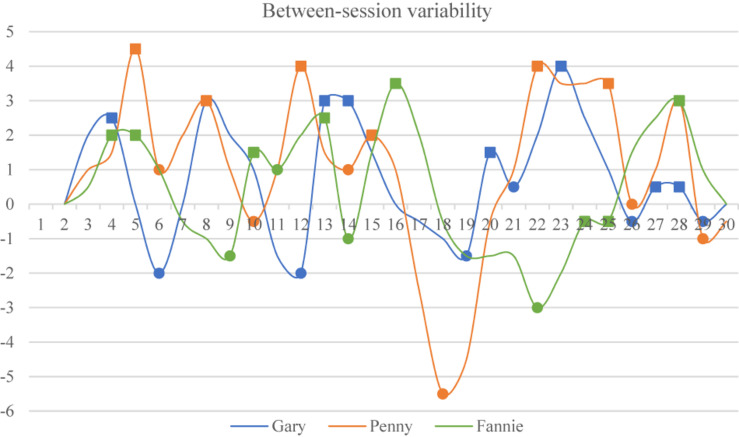
Inter-individual between-session variability.

Penny demonstrated the highest degree of variability among the three learners as shown in aforementioned results. Variability is inherent in any complex system, and a close enquiry of this variability can help to detect how a system changes from one phase to the next (De Bot et al., 2005). In this case, it is suggested that variations demonstrated by learners with a higher degree of variability might not be isolated jumps, but rather a continuous developmental process (van Dijk et al., 2011; Verspoor et al., 2017). In order to illustrate this, the between-session variability is first illustrated in Figure 3, and then analyzed through Monte Carlo analysis.

We started by identifying the moving averages over two observations of learners’ listening performance, and then calculated the maximum distances between two data points (e.g., between the first and second observations, the first and third, the first and fourth, and finally the first and sixth, followed by the second and third, second and fourth, and so on). Thus, the original data from the three learners’ between-session variability are presented in Figure 3. It can be seen from the diagram that the three learners demonstrated different levels of variability with different numbers of peaks (marked by squares) and valleys (marked by circles). Specifically, the degree of Gary’s between-session variability was between −2 and +4 points. Fannie’s variability ranged between −3 and +4 points, and Penny demonstrated the highest degree of between-session variability, between −5.5 and +4.5 points.

Aiming to identify the differences among the three learners’ between-session variability, and to ascertain whether Penny was indeed the one with the highest degree of variability, Monte Carlo analyses were arranged to resample the original data related to the participants’ between-session variability. It was supposed that if the original data were resampled, the maximum values would be the same as in our resampling set. Thus, the maximum values that each learner demonstrated were first selected to form the empirical testing criterion. In this case, the original data from each participant were resampled as a new model for Monte Carlo analyses (Verspoor, 2017). It is important to note that a new set is randomly drawn from the original pool as resampled data in each simulation, which implies that we resampled our data with replacement in Monte Carlo analyses 5000 times (for more information see van Dijk et al., 2011; Verspoor, 2015, 2017). Then we ran three separate Monte Carlo analyses for Gary, Penny and Fannie with 5000 simulation steps. The results are presented in Table 1.

**TABLE 1:** Results of between-session variability (Monte Carlo analyses).

Participants	Significance value
Gary	0.012*
Penny	0.002**
Fannie	0.023*

*Variability is significant at the 0.05 level. **Variability is significant at the 0.01 level.

It can be seen from Table 1 that the significant values for the Monte Carlo analyses for Gary and Fannie were 0.012 and 0.023, respectively, which reached significance (*p* ≤ 0.05). This means that the peaks that Gary and Fannie demonstrated are not coincidental fluctuations and might happen again in their future learning process. Another significant result was found in Penny’s between-session variability (*p* = 0.002, *p* ≤ 0.01), which shows that Penny would probably re-experience peaks similar to those that she showed in this study, which is a possible future developmental tendency.

These results show that inter-individual variability existed within all three learners’ listening developmental trajectories. The three learners’ fluctuations were not isolated, and Penny is confirmed as the one with the highest degree of between-session variability. Thus, it appears that no individual behaves like the average values depicted in traditional SLD studies (Larsen-Freeman, 2019), with each individual demonstrating diverging patterns within their own developmental trajectory, as well as showing disproportionate amounts of variability when they move from one stage to the next (Verspoor, 2017). This is the intra-individual variability that our next section concentrates on.

### Intra-Individual Variability

Intra-individual variability is worthy of exploration from a CDST perspective (Verspoor, 2017; Larsen-Freeman, 2019). Different kinds of variability patterns are expected to characterize different kinds of learning trajectory, and illustrate various developmental changes (Verspoor et al., 2008). Concerning each individual learner’s subjective and conscious experiences, IPA was carried out by researchers in four stages across the participants’ transcribed interviews and their self-reflections.

In the first stage of the IPA, we noted the motivation diversity among Gary, Penny, and Fannie. Specifically, Gary was determined to further his postgraduate study in America and studied TOEFL during his 4-year university life. Penny did not plan her postgraduate study until she was inspired by a senior’s speech, starting with IELTS practice at that point. With the ultimate aim of getting a decent and high-pay job, Fannie worked hard at university and finally achieved her goal.

The second stage of IPA involved investigating each learner’s unique learning route and finding evidence (e.g., internal feelings, external stimulations) to confirm our previous statistical results about the participants’ non-linear developmental trajectories. In the third stage of the IPA we worked with this evidence in detail, concerned with and comparing participants’ different learning experiences and similar circumstances. Thus, in the final stage a number of central themes of inter-individual variability emerged, namely constant change, internal factors (e.g., initial conditions), external factors, and attractor and repellor states.^2^

**FIGURE 4:**
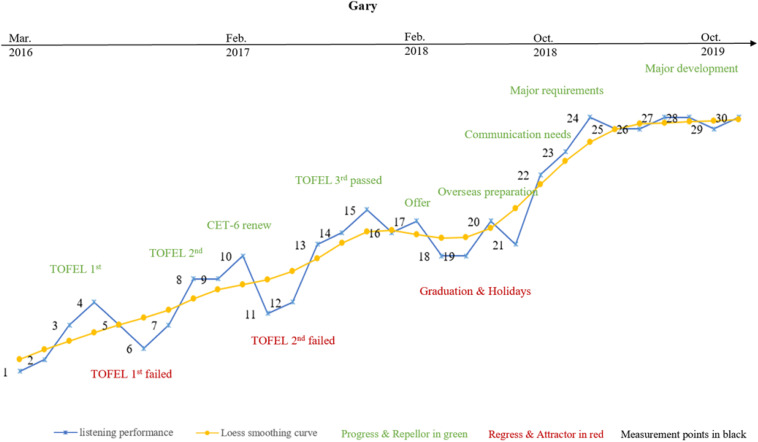
Gary’s variability patterns and listening trajectory.

**FIGURE 5:**
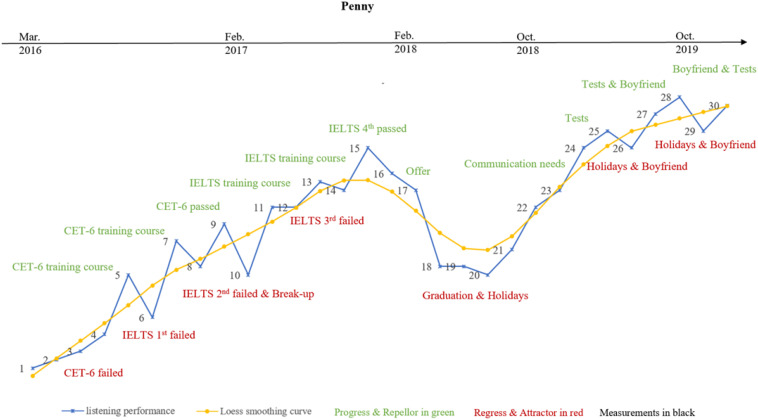
Penny’s variability patterns and listening trajectory.

**FIGURE 6:**
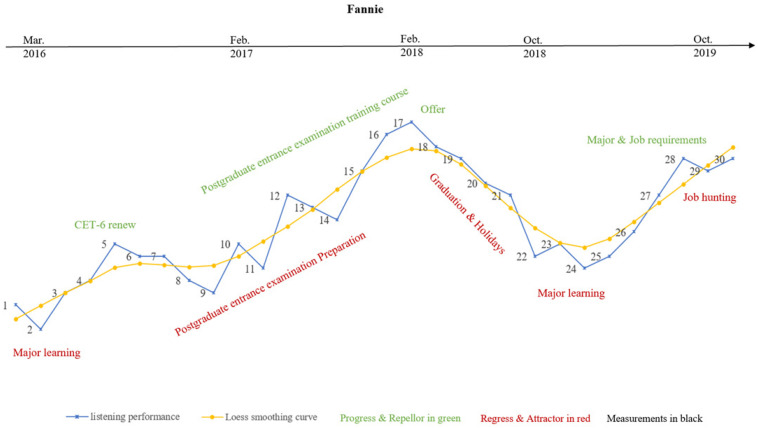
Fannie’s variability patterns and listening trajectory.

**FIGURE 7:**
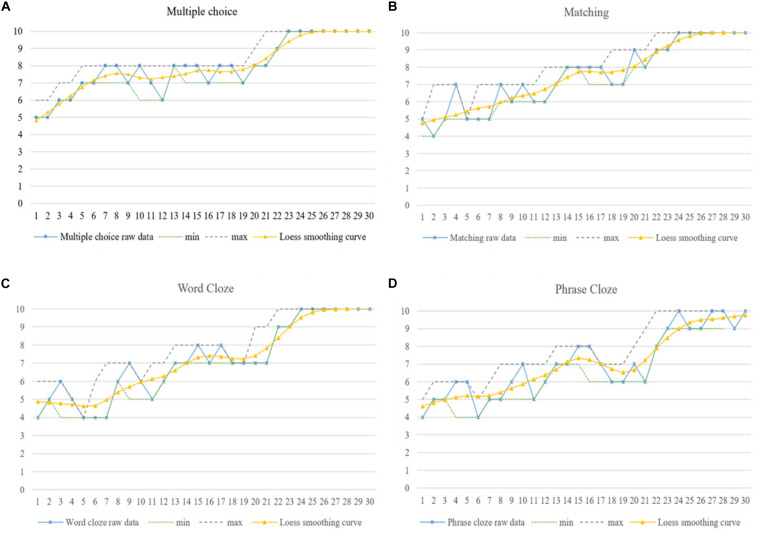
Gary’s dynamic listening developmental trajectories in four aspects of **(A)** Multiple choice, **(B)** Matching, **(C)** Word Cloze and **(D)** Phrase Cloze.

**FIGURE 8:**
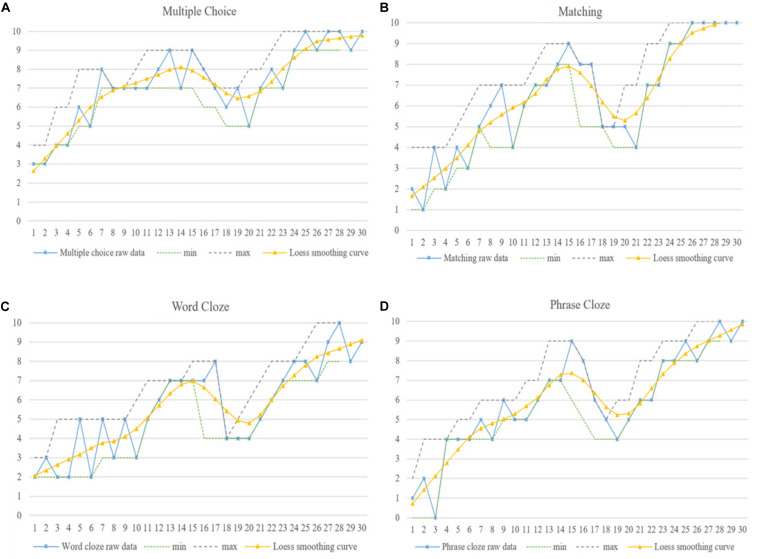
Penny’s dynamic listening developmental trajectories in four aspects of **(A)** Multiple choice, **(B)** Matching, **(C)** Word Cloze and **(D)** Phrase Cloze.

**FIGURE 9:**
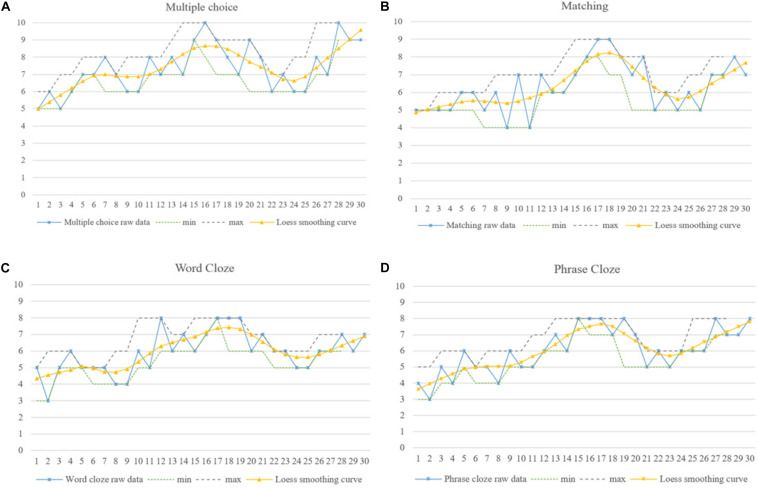
Fannie’s dynamic listening developmental trajectories in four aspects of **(A)** Multiple choice, **(B)** Matching, **(C)** Word Cloze and **(D)** Phrase Cloze.

There must be reasons to explain why participants’ listening developed iteratively between “attractor states” and “repellor states.” This kind of state fluctuations cannot be predicted, but it may be inferred by learners’ initial conditions, or accompanied by various constant changes throughout the developmental trajectory (De Bot and Larsen-Freeman, 2011; Hiver, 2015). Aiming to clearly illustrate these emergent themes in the learners’ listening trajectories, we summarize the participants’ IPA results with the raw data from their listening performance in Figures 4–6, supplemented with the smoothed Loess curves to clarify the developmental tendency of the three participants’ learning processes in four individual listening sections in Figures 7–9.

#### Gary’s Variability Pattern and Learning Trajectory

As shown by the IPA analysis, intra-individually, TOEFL is the main factor affecting Gary’s progress as well as his setbacks during the first stage of listening development (M-1 to M-10). Gary commented in his first reflection that the “TOEFL test is a very intense activity and is quite different from CET 4 and 6 tests. There are more professional lecturers with academic terminologies in the listening test, which blocks me to capture the specific information and infer the pragmatic meaning. I failed my first TOEFL test, and made mistakes ceaselessly. I feel frustrated!” It can be seen from Figures 7A–D that Gary performed differently in the four sections, and noticeable regressions were revealed in his performance in matching and cloze questions from M-4 to M-7. This proved that Gary’s “initial condition” in doing TOEFL tests caused him difficulty in this beginning stage of his listening development, which might constitute his first attractor state (De Bot and Larsen-Freeman, 2011; van Dijk et al., 2011).

After the second stage of listening development (M-11 to M-15), however, he overturned his statement in the previous interview, and reported: “TOEFL is indeed a challenge – I did more practice and seemed to find ways to conquer it. I feel excited and would definitely keep working on it, even if I did not get an ideal score in my recent test [referring to his second TOEFL test]. I will achieve my ideal results [referring to application requirements for his postgraduate learning] next time! I am confident about myself!” In fact he achieved a very good score in his third TOEFL test (M-15), and was accepted by his dream university in February 2018 (around M-17). It can be seen from Figure 4 that the smoothed Loess curve depicted a general upward trend, even if there were different levels of fluctuation in his listening development from M-11 to M-15. Attributing his improvement to his determination and confidence, he devoted more attention, time and energy to the TOEFL tests, and his performance in the four individual listening sections also showed upward development and progress. Thus, self-confidence proved to be an important internal element in the dynamic process of Gary’s staged success, confirming the results of some previous studies (De Bot and Larsen-Freeman, 2011; van Dijk et al., 2011).

It can be seen from the smoothed Loess curve in Figure 4 that Gary’s listening showed a slightly downward trend after M-15, which was confirmed by his report that “I did not do any practice recently because I was busy with the graduation project from February to June 2018, and enjoying the summer vacation with my friends” (from M-15 to M-20). IPA results and his listening performance in each section confirmed that less exposure and meaningful use of the target language can lead to the development of “attractor states” (De Bot and Larsen-Freeman, 2011; van Dijk et al., 2011). Gary’s overseas learning experience motivated further listening improvement from M-21 to M-30, which can be seen from the smoothed Loess curves for Gary’s overall listening in Figure 4 and the narrower bandwidths of the min–max graphs for Gary’s listening performance in each section in Figure 7. Moreover, a much more balanced performance was detected in Gary’s listening scores for each individual section.

The further IPA results show that Gary focused on daily communication, learning different subjects and career planning simultaneously from M-22 onward, rather than focusing on TOEFL tests only as he had done in earlier initial phases (e.g., M-1). Gary concluded: “I did not adapt to the overseas life several months ago, which drove me crazy to handle both the living trifles and learning myself. But I tried to manage them and sorted everything out. Look at me now – I am happy to study and live here and I have many friends from other countries. I also did pretty well in my postgraduate program and was not afraid of doing presentations anymore. All of these helped me to expand my knowledge about oral idioms as well as terminologies in my field of study as a student majoring in Statistics, which I felt obscure and strange when preparing for my TOEFL test in 2016. Now, I feel motivated and energized to continue my learning and life here. I plan to work or apply for Ph.D. study when I get my Master’s degree. Wish me good luck!” Gary’s listening performance from M-27 to M-30 indicated that he gradually adapted to his new surroundings. There might be some downturns in Gary’s future learning, but those will be temporary stages in his upward listening developmental trajectory over the long term.

#### Penny’s Variability Pattern and Learning Trajectory

Penny’s listening trajectory and smoothed Loess curve are presented in Figure 5. It can be seen that she experienced more fluctuations and developed into attractor and repellor states more frequently during the 43-month observation. It should be noted that Penny’s IPA results showed that Penny assessed herself as an emotional person, and she reported although she was driven by different tests (e.g., CET-6 and IELTS), she was motivated to undertake all of these by the expectations of her parents; this is quite different from Gary, who aimed to perfect himself.

Penny explained, “I definitely should work hard, but my parents seem much more eager than me. They expected higher scores, treasured overseas learning opportunities, which motivated me to work hard and attend extra language training classes because I did not want to let them down.” Thus, Penny made continuous progress from M-1 to M-15, shown by the smoothed Loess curve depicted in Figure 5. However, it can be seen from her overall listening performance in Figure 5 and the large bandwidths of the min–max graphs in Figures 8A–D that Penny’s listening progress was full of ups and downs. Especially, there was a rapid decrease in her listening during M-10. The reasons were detected from her IPA results: “the unstable and unexpected relationship with my boyfriend drove me crazy. I can focus on my study when we are in a good relationship; otherwise, I cannot concentrate and feel upset if we fight. By the way, we broke up finally in the last few days” (around M-10). It may be inferred from Penny’s reflections that the instability of her emotional relationship affected her listening performance some of the time, and this might result in her unexpected “attractor and repellor states” (Larsen-Freeman, 2006). This interpretation supplemented our statistical results that Penny demonstrated the highest degree of variability, and her “peaks” were not isolated ones in her listening development.

Moreover, “graduation and summer holiday” were reported by Penny as additional major contributors to her listening development, and to her regression particularly. She emphasized in her reflections: “I did not continue with any training courses or do any listening practice recently because I was fully occupied by my graduation thesis [around M-16 to M-18], and there were no more tests I had to take. I just wanted to relax during the summer holidays [from M-18 to M-21] before the postgraduate learning starts in September [2019].” Her listening performance dropped rapidly from M-16 as shown by the smoothed Loess curve presented in Figure 5; this is also confirmed by the sudden decrease in the smoothed Loess curves in each listening section (see Figure 8). An obvious “U” shape was shown in her listening performance from M-15 to M-20, and Penny’s listening developed into another “attractor state.”

Further IPA results identified “new environment,” “communication needs,” and “academic pressure” as reasons for Penny’s “repellor state” that started at M-20. Penny reported: “the new environment of my postgraduate study in Hong Kong brought me both pressures and opportunities. I must devote myself to practicing language and completing the assignments required of students who major in Statistics simultaneously.” It can be seen from the smoothed Loess curves in both Figures 5, 8 that Penny made continuous and rapid progress from M-20 to M-25, demonstrating step-like achievement in each listening section. Moreover, “parents expectation/encouragement,” “emotional conditions,” and “sensitive and impulsive personality” were identified as important triggers for her fluctuations from M-25 to M-30, when she concluded: “I am a sensitive, impulsive and impressionable person who is easy to be influenced by others. Thus, my listening fluctuations probably represented my emotional fluctuations with my parents or boyfriend. Honestly, I did not plan too much, but I am satisfied with my current situation. I am not sure about the future, but I will definitely work hard to pursue a better life.” The smoothed Loess curve in Figure 5 demonstrates an increasing trend in Penny’s final stage of listening development (M26 to M30), and the smaller bandwidth of the min–max graphs in Figure 8 also indicate her better performance in each listening section. Thus, it may be inferred that Penny will experience an upward trend in her ongoing listening developmental trajectory, with unpredictable fluctuations because of the aforementioned factors. This might be one of the reasons why Penny demonstrated the highest between-session variability, as indicated by the quantitative results.

#### Fannie’s Variability Pattern and Learning Trajectory

Statistical results in the previous section showed that Fannie’s listening performance was significantly different from Penny’s (*p* = 0.05), but not from Gary’s (*p* = 0.09). Furthermore, the variation results indicated that all the peaks in Fannie’s listening development would happen again (*p* = 0.023). The smoothed Loess curves showing Fannie’s listening developmental trajectory are presented in Figures 6, 9, and the qualitative IPA results showed that Fannie was highly concerned about “test scores” for both her listening and major subjects, which were prerequisites for her getting a “decent job.” Thus, “attentional competence” was reported by Fannie as a unique feature in her listening development, because she had to maintain a balance between practicing my L2 listening skills and studying courses as a Software Design major. Evidence for this can also be found in Penny’s listening performance as depicted in Figure 6, where there is a significant decrease at M-2. At this time Penny also reflected: “I was preparing for a software designing test related to my major and did not have much time for practicing listening recently.”

Moreover, this competition between learning subjects in my major field of concentration as a university student and practicing listening was a special reason for Fannie’s “attractor and repellor states” during her listening development, as shown in Fannie’s listening performance on each individual section (from M-1 to M-10 approximately) in Figure 9. During this period, Fannie reflected: “I started to prepare the examination for postgraduate learning recently, which means I have to review courses required of me as a student of that major and English simultaneously. Much more time and energy is needed. Sometimes I feel upset because of the chaos.” Fannie’s listening demonstrated an upward trend with fluctuations at varying degrees from M-11 to M-17, which are represented by the smoothed Loess curves in Figures 6, 9. Fannie’s IPA results showed that “peer/group learning” and “roommates discussion” were reported to be effective strategies for enhancing her language proficiency. Fannie explained: “I joined a learning group with my roommates. We took classes and completed model tests together, then discussed our learning experiences and provided feedback for each other. I felt more energetic and motivated than practicing alone. I benefited so much from our learning group!” Fannie’s reflections support the claim that individuals possess a self-organizing system, and they might make adjustments when faced with novel circumstances or learning tasks (De Bot and Larsen-Freeman, 2011).

Similar to Gary’s and Penny’s developmental trajectories, a rapid decrease was detected in Fannie’s listening performance from M-17 to M-22, because of graduation and summer holidays. During this period Fannie’s listening seemed to develop into a “repellor state.” This is understandable, since participants are not perpetual motion machines but living persons who sometimes needs a rest. Fannie went on to make continuous progress and demonstrate less fluctuation in her follow-up measurements (M-23 to M-30), as also shown in Figures 6, 9, indicating her relatively steady listening developmental trajectory.

Further IPA results also confirmed that finding a decent job was Fannie’s ultimate goal in her Bachelor’s and Master’s degree learning experiences, stimulating and guiding her during the 43-month period of observation. Thus, we may infer that Fannie’s learning process might end in the future if she is offered a good and high-pay job. However, she will continue to make efforts to development her listening competence if the job is offered by an international company. This might be the reason why Fannie’s “peaks” were not coincidental ones like the statistical results in our previous analysis.

#### Intra-Individual Variability and Development

The smoothed Loess curves in Figures 4–6 depict that each learner demonstrated an overall upward trend in their listening development. Meanwhile, the min–max graphs in Figures 7–9 confirmed the three learners’ performance imbalance in each section and their constant dynamic changes. Taken together, these results indicate that phase transitions between progress and regression, i.e., between “attractor and repellor states,” may be an inherent characteristic of learners’ listening developmental pattern. Each learner may demonstrate completely different learning processes and variability patterns even if they were exposed to similar circumstances (Larsen-Freeman, 2006; Bulté and Housen, 2014; Chan et al., 2015).

**FIGURE 10:**
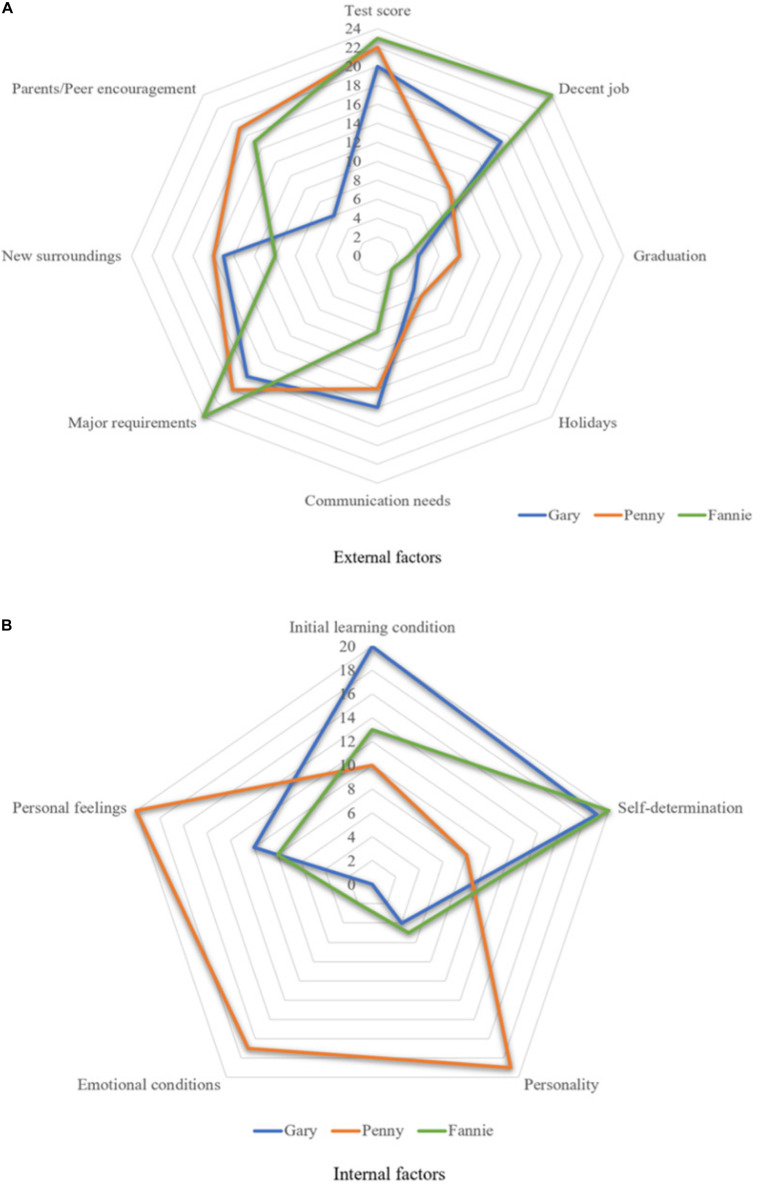
Intra-individual variability patterns influenced by **(A)** external and **(B)** internal factors.

The IPA explored the participants’ listening developmental process, and the findings revealed that there were various factors playing important roles in learners’ phase transitions between “attractor and repellor states.” According to the categorization by Larsen-Freeman (2017), these elements may be classified into two different variability patterns: One concerns environmental-related “external factors” (e.g., test scores), while the other is personal-related “internal factors” (e.g., emotional conditions). The diagrams in Figure 10 illustrate how frequently these various reasons were invoked by participants in their self-reflections (24 times in total) during the longitudinal observations.

It can be inferred from participants’ reflections as well as the diagrams that Gary and Fannie were learners who were mainly affected by “external factors,” while Penny was a sensitive learner with more “internal factors,” especially her personality, emotional conditions, and personal feelings. This might be a reason why she demonstrated the highest degree of intra-individual variability. Meanwhile, these different variability patterns ensure that participants will fluctuate continuously along their listening development trajectory, as our statistical results confirmed.

Thus, it may be concluded that each learner is a complex self-organizing system whose learning can be affected by both environment-related “external factors” and personal-related “internal factors,” although to varying degrees. The current findings concur with Chang and Zhang (2020) results that the dynamic ebbs and flows of EFL listeners’ motivation were affected by both internal (e.g., learners’ goals) and external (e.g., learning contexts) factors. It is vital to consider intra-individual differences within the developmental process, particularly to illustrate ongoing and diverging patterns and identify state transitions. This would be an effective way to identify personal learning traits and explore the central elements of such a developing system (Larsen-Freeman, 2017, 2019). Finally, it should be noted that even if participants were exposed to similar circumstances, their listening developmental trajectories will be completely different, which again shows the necessity of investigating variability, both within and between individuals (Verspoor et al., 2017).

## Conclusion

The current longitudinal study traced three learners’ dynamic change in L2 listening development over a period of 43 months, investigating individual differences in inter-individual variability in learning trajectories and intra-individual variability among one another, using min–max graphs, smoothed Loess curves, Monte-Carlo analysis, and IPA. The different bandwidth of the min–max graphs revealed that the three learners demonstrated different developmental trajectories. This inter-individual variability was further confirmed by the statistical results of Monte-Carlo analysis, where Penny’s listening developmental trajectory was found to be significantly different from those of Gary (*p* = 0.002) and Fannie (*p* = 0.05).

Moreover, results related to between-session variability showed that Penny’s developmental trajectory displayed higher variability than those of her two counterparts during the 43-month observation. Further statistical results confirmed that the constant fluctuations in her listening trajectory were not likely to be coincidental (*p* = 0.002). The reasons for this were explored through IPA using retrospective interviews and learners’ self-reflections, and the results showed that participants demonstrated significant intra-individual variability in their individual process of listening capacity development, which were shown in their own variability patterns.

As shown by the IPA results in Figure 10, the variability patterns in listening development were influenced by various factors to varying degrees, including environmental-related “external factors” (e.g., test scores, new surroundings, and holidays) and personal-related “internal elements” (e.g., emotional conditions and personal feelings). Both were found to play critical roles in the development of listening ability and its attrition. Therefore, L2 learners’ listening performance in the four sections further suggests that intense fluctuations and significant variability tended to occur in the proximity of a phase transition, and regression in “attractor states” could, to some extent, predict future progress in “repellor states.” On one hand, intra-individual variability permits flexible and adaptive behavior. On the other hand, differences in performances between individuals indicate inter-individual variability in the developmental trajectory, even if participants are exposed to similar circumstances, as highlighted by Larsen-Freeman (2020).

In addition to the contribution that this study has intended to make to the theoretical and methodological knowledge of L2 listening research from a complex dynamic systems perspective, as discussed above, we think that the findings of the current work can also help to enrich our understanding of variability; namely, variability is associated with long-term change and shown by variability patterns in developmental trajectories. The CDST methods adopted in the current study might have various applications. Firstly, these different methods (e.g., the min–max graphs) can be adopted in tracking and documenting EFL learners’ progress in learning new languages, where not only listening but also speaking, reading and writing skills can be checked. Secondly, with the help of Monte-Carlo analysis as well as smoothed loess curves, learners’ dynamic development in different aspects of language learning could be visualized and tracked, which would assist teachers in previewing learners’ language development. Finally, compared with the traditional research design of pre- and post-test, these CDST methods and data analysis tools as used in our study can be more effective in recording and noticing learners’ complex learning processes. It is hoped that the current results will be useful for practitioners taking a dynamic systems approach to teaching and assessing students’ listening performance, incorporating dynamic developmental characteristics into listening practices and adjusting pedagogies for teaching listening, designing materials and creating assignments to cater for students’ developmental learning characteristics and requirements.

It is necessary to point out that this study only focused on three participants. Considering that different learners may vary in the way that they approach listening (Larsen-Freeman, 2009), any generalization of the findings of this study to the development of the English listening performance of other learners should be undertaken with caution. Follow-up studies will benefit from employing diverse research methods to explore learners’ performance in other aspects of English learning, or with other variables. Following De Bot and Larsen-Freeman (2011) suggestion that taking a holistic view of the CDST approach is necessary, it would be of great interest to explore learners’ dynamic trajectories and variations in L2 performance alongside other variables, and investigate how different variables may interact with other variables within this diverse and dynamic system.

## Data Availability Statement

The original contributions presented in the study are included in the article/Supplementary Material, further inquiries can be directed to the corresponding author.

## Ethics Statement

The studies involving human participants were reviewed and approved by The University of Auckland Human Ethics Committee. The patients/participants provided their written informed consent to participate in this study.

## Author Contributions

PC conceived of the initial idea, fine-tuned by LZ. PC designed the study, collected and analyzed the data, and drafted the manuscript. LZ revised and proofread the manuscript. LZ finalized the draft for submission as the corresponding author. Both authors have approved the submission.

## Conflict of Interest

The authors declare that the research was conducted in the absence of any commercial or financial relationships that could be construed as a potential conflict of interest.

## Footnotes

1 www.ielts.org

2 The “attractor” is like a ball rolling over on the surface such as that of the moon, partly smooth, partly characterized by holes and mountains. It will be attracted to the holes and repelled by the mountains (De Bot and Larsen-Freeman, 2011, p. 15).
