# Supplementary material for: A CDST Perspective on Variability in Foreign Language Learners’ Listening Development
Source: Front Psychol. 2021 Feb 3;12:601962. doi: 10.3389/fpsyg.2021.601962 (PMC7887325; doi:10.3389/fpsyg.2021.601962)
Supplement: Supplementary file 1 [file Data_Sheet_1.docx]

**APPENDICES**

**Appendix I: Listening comprehension test**

**SECTION 1  *Questions 1-10***

***Questions 1-5***

Complete the notes below.

Write **NO MORE THAN THREE WORDS** for each answer.


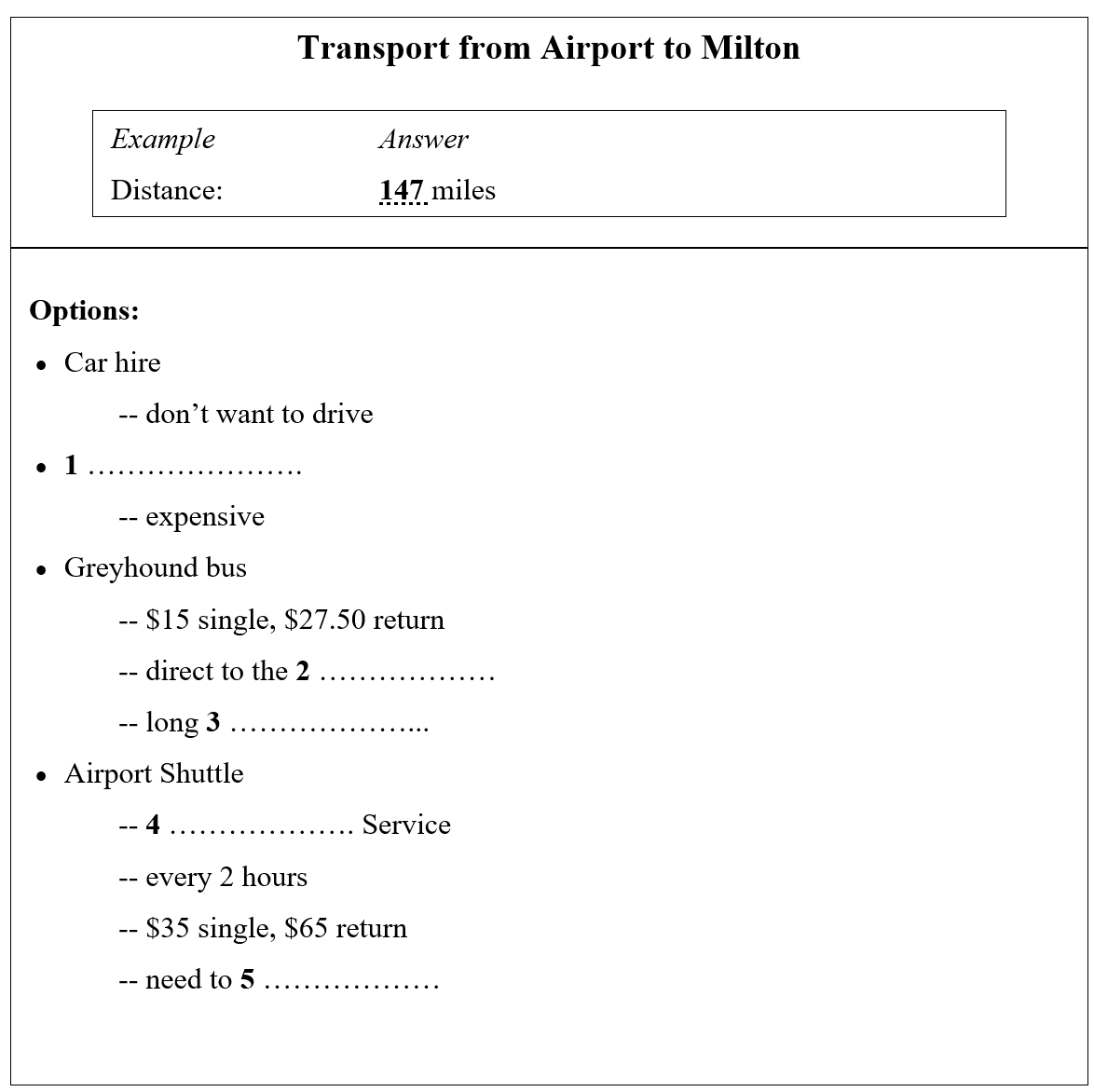


***Questions 6-10***

Complete the booking form below.

Write **ONE WORD AND/OR A NUMBER** for each other.


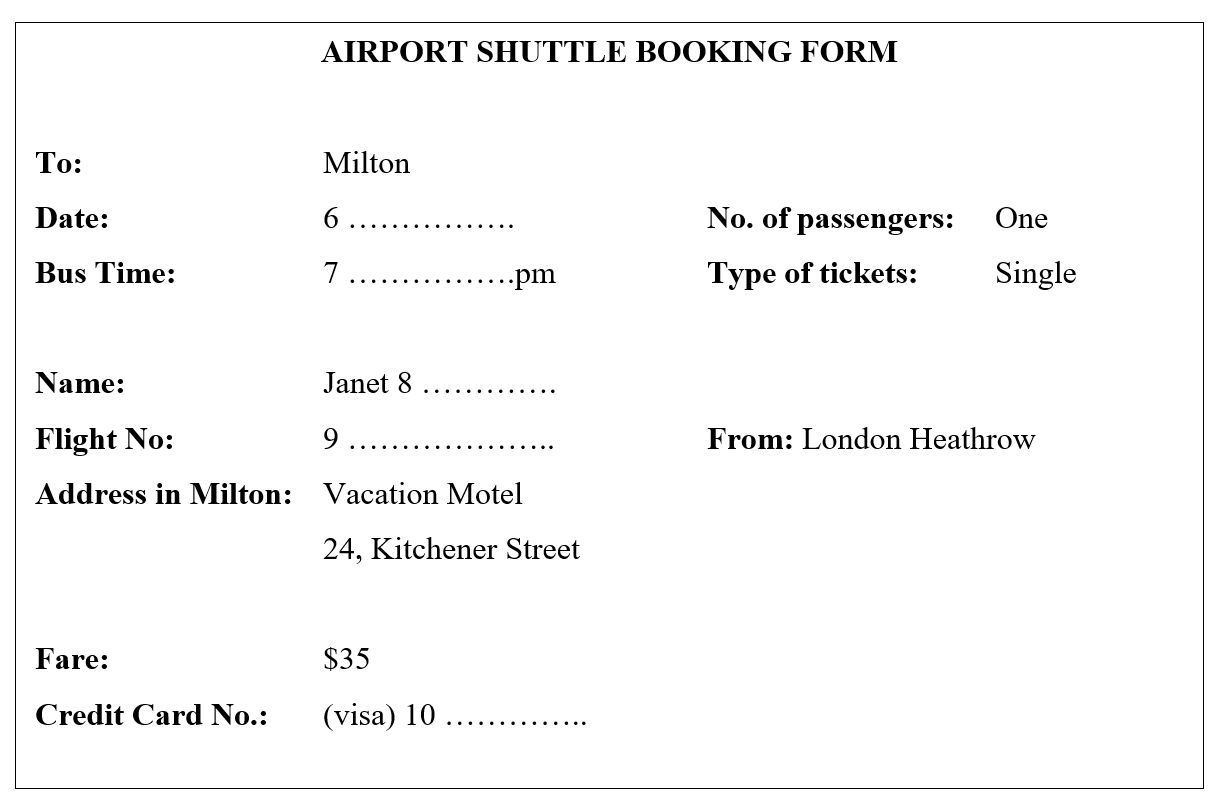


***SECTION 2 Questions 11-20***

***Question 11-16***

Choose the correct letter, **A, B** or **C**.

11. PS Camping has been organising holidays for

A. 15 years. B. 20 years. C. 25 years.

12. The company has most camping sites in

A. France. B. Italy. C. Switzerland.

13. Which organised activity can children do every day of the week?

A. Football. B. Drama. C. Model making.

14. Some areas of the sites have a ‘no noise’ rule after

A. 9.30 p.m. B. 10.00 p.m. C. 10.30 p.m.

15. The holiday insurance that is offered by PS Camping

A. can be charged on an annual basis.

B. is included in the price of the holiday.

C. must be taken out at the time of booking.

16. Customers who recommend PS Camping to fiends will receive

A. a free gift. B. an upgrade to a luxury tent. C. a discount.

***Questions 17-20***

What does the speaker say about the following items?

Write the correct letter, **A, B** or **C**, next to questions 17-20.

**A. They are provided in all tents.**

**B. They are found in central areas of the campsite.**

**C. They are available on request.**

17. barbecues ……….

18. toys ……….

19. cool boxes ……….

20. mops and buckets ……….

**SECTION 3 Questions 21-30**

***Questions 21-23***

Complete the notes below.

Write **ONE WORD ONLY** for each answer.


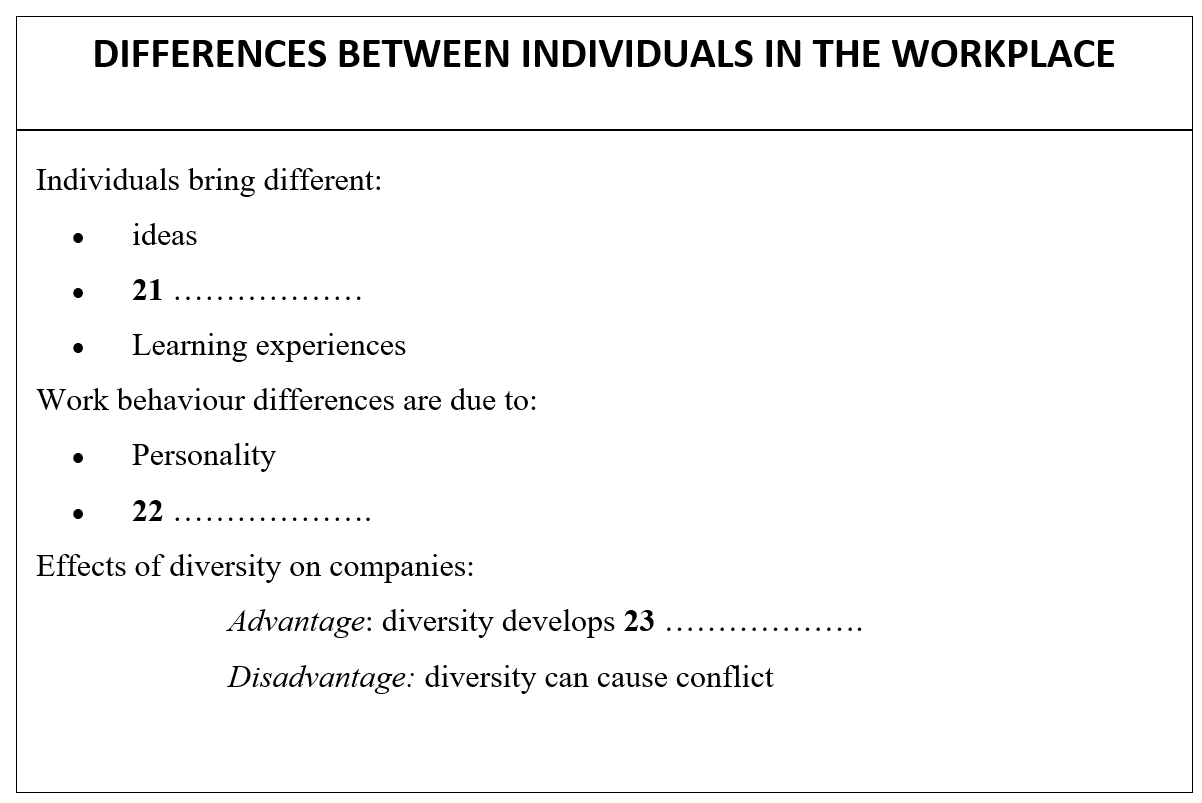


***Questions 24-27***

Choose the correct letter, **A, B** or **C**.

24. Janice thinks that employers should encourage workers who are

A. potential leaders.

B. open to new ideas.

C. good at teamwork.

25. Janice suggests that mangers may find it difficult to

A. form successful groups.

B. balance conflicting needs.

C. deal with uncooperative workers.

26. Janice believes employers should look for job applicants who

A. can think independently.

B. will obey the system.

C. can solve problems.

27. Janice believes managers should

A. demonstrate good behaviour.

B. encourage co-operation early on.

C. increase financial incentives.

***Questions 28-30***

Complete the sentences below.

Write **ONE WORD ONLY** for each answer.

28. All managers need to understand their employees and recognise their company’s ………...

29. When managing change, increasing the company’s …...…..……may be more important than employee satisfaction.

30. During periods of change, managers may have to cope with increased amounts of …………….. .

**SECTION 4 Questions 31-40**

***Questions 31-35***

Complete the notes below.

Write **ONE WORD ONLY** for each answer.


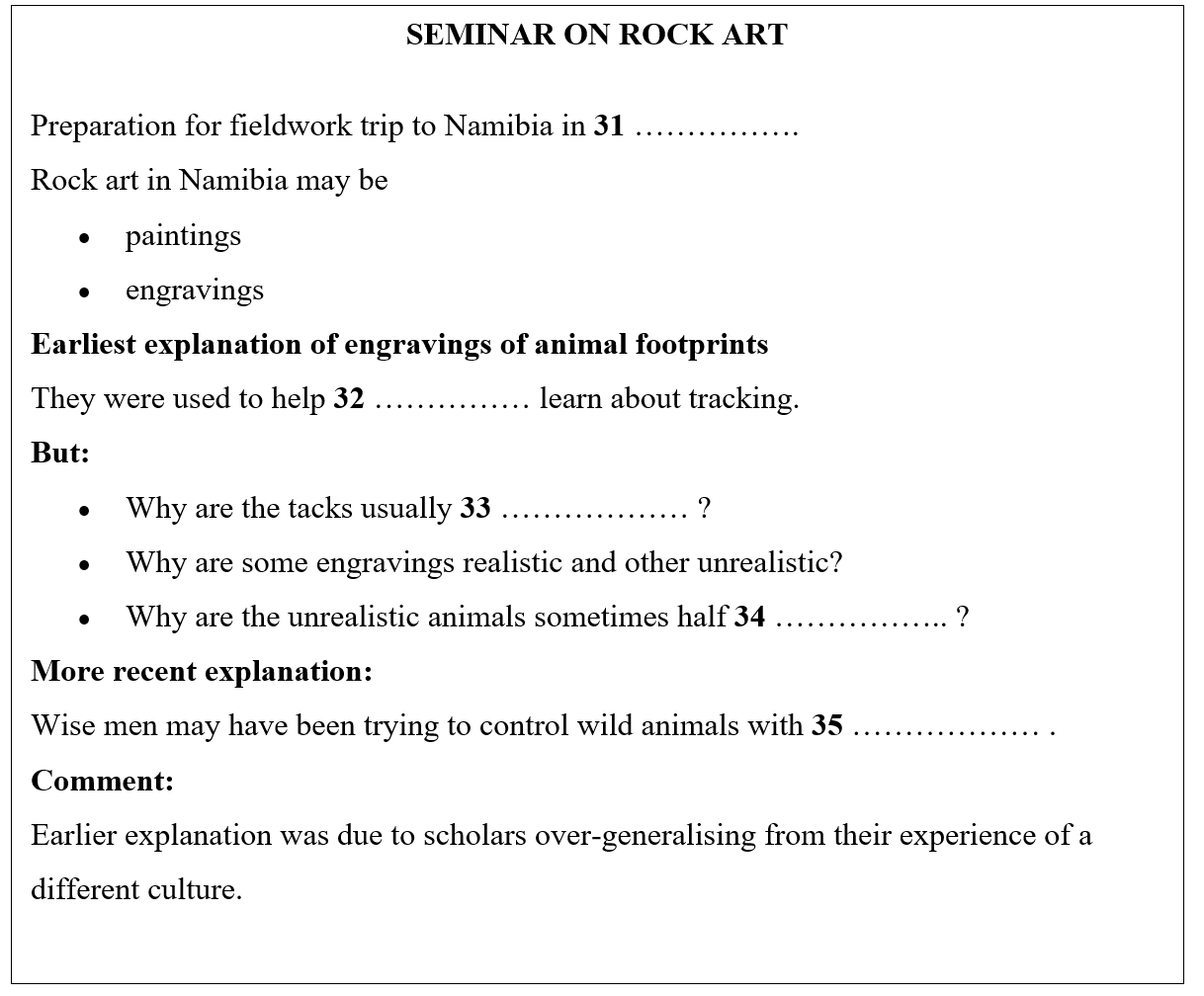


***Questions 36-40***

Complete the sentences below.

Write **ONE WORD ONLY** for each answer

36. If you look at a site from a ………….. , you reduce visitor pressure.

37. To camp on a site may be disrespectful to people from that ……………. .

38. Undiscovered material may be damaged by ………………… .

39. You should avoid ……………. or tracing rock art as it is so fragile.

40. In general, your aim is to leave the site …………….. .

**Appendix II: Retrospective interview guide**

|  | ***Interview questions*** |
| --- | --- |
| 1. | Why do you want to learn and practice your listening during the last month? |
| 2. | What do you think of your listening ability compared with the last measurement? |
| 3. | Do you have any difficulties with recent listening practices? What are they? |
| 4. | What are the main factors that influence your listening performance? (e.g., pronunciation, speed rate, vocabulary, content etc.) |
| 5. | What do you usually do to practice your listening after class? What kind of materials do you usually use? |
| 6. | Which section of the listening test is the most demanding or difficult part to you? |
| 7. | If you cannot follow the listening material, what do you usually do? |
| 8. | Did you use some strategies to conquer the nervousness and confusions while listening? |
| 9. | Do you have any special aims when practicing listening recently (e.g., exams, interests)? |
| 10. | How do you evaluate your listening currently? Did you make progress? Why do you think so? |
| 11. | How is your recent emotional and life situation, will these aspects influence your listening practices? |

**Appendix III: Self-reflection prompts**

|  | ***Prompts for self-reflection*** |
| --- | --- |
| 1. | How much did I understand the materials? What were the problems in understanding the materials in the four individual listening sections (e.g., vocabulary, accent, translation, memory capacity, idioms, etc.) |
| 2. | What were the materials I listened to in this week? (e.g., English movies, listening comprehension tests or exercises, dictations, etc.) |
| 3. | What were the strategies I usually use while listening? (e.g., translation, take notes, inferencing, guessing, elaboration, key words, etc.) |
| 4. | What did I do to practice my listening after class? (e.g., enlarge the vocabulary, learn about pronunciation skills, listen to authentic English news, movies, or songs, etc.) |
| 5. | What is my recent goal in practicing listening? |
| 6. | What did I do to understand as much information as possible while listening? (e.g., recover my concentration when my mind wanders, encourage myself when nervous, give up or keep listening when I feel difficult, etc.) |
| 7. | How is my listening recently? Did I make progress? Did I meet my goals for listening? |
| 8. | What am I going to do with the listening practice in the next seven weeks? |
| 9. | Are there any things that might boost or weaken my interests in listening? (e.g., emotional conditions; learning subjects in my major field; friends and parents influences, etc.) |
| 10. | Did I make progress in reading, vocabulary, pronunciation skills, or grammar learning recently? Did they contribute to my listening proficiency? |
